# A multi-level, multi-component obesity intervention (Obesity Prevention and Evaluation of InterVention Effectiveness in NaTive North Americans) decreases soda intake in Native American adults

**DOI:** 10.1017/S1368980020001172

**Published:** 2022-03

**Authors:** Leslie C Redmond, Brittany Jock, Fariba Kolahdooz, Sangita Sharma, Marla Pardilla, Jacqueline Swartz, Laura E Caulfield, Joel Gittelsohn

**Affiliations:** 1 Department of Dietetics and Nutrition, School of Allied Health, College of Health, University of Alaska Anchorage, 3211 Providence Dr., PSB 146H, Anchorage, AK 99508, USA; 2 McGill University, Quebec, Canada; 3 Division of Endocrinology, Department of Medicine, University of Alberta, Edmonton, Alberta, Canada; 4 Department of International Health, Bloomberg School of Public Health, Johns Hopkins University, Baltimore, MD, USA

**Keywords:** Obesity, Intervention, Native American, Nutrition, Sugar-sweetened beverages

## Abstract

**Objective:**

To evaluate the impact of a multi-level, multi-component (MLMC) adult obesity intervention on beverage intake in Native American adults living in five geographically and culturally diverse tribal communities.

**Design:**

A 14-month, community-randomised, MLMC design was utilised, with three communities randomised to Intervention and two communities randomised to Comparison. FFQ were administered pre- and post-interventions, and difference-in-differences (DiD) analysis was used to assess intervention impact on beverage intake.

**Setting:**

The intervention took place within food stores, worksites, schools and selected media outlets located in the five communities. Key activities included working with store owners to stock healthy beverages, display and dispersal of educational materials, support of policies that discouraged unhealthy beverage consumption at worksites and schools and taste tests.

**Participants:**

Data were collected from 422 respondents between the ages of 18 and 75 living in the five communities pre-intervention; of those, 299 completed post-intervention surveys. Only respondents completing both pre- and post-intervention surveys were included in the current analysis.

**Results:**

The DiD for daily servings of regular, sugar-sweetened soda from pre- to post-intervention was significant, indicating a significant decrease in Intervention communities (*P* < 0·05). No other changes to beverage intake were observed.

**Conclusions:**

Large, MLMC obesity interventions can successfully reduce the intake of regular, sugar-sweetened soda in Native American adults. This is important within modern food environments where sugar-sweetened beverages are a primary source of added sugars in Native American diets.

Excessive energy intake in the form of energetic beverages is an important consideration in the prevention of overweight and obesity. Sugar-sweetened beverages (SSB), in particular, are the single largest source of added sugars in the typical American diet and contribute significantly to the nationwide obesity epidemic^(1)^. This is especially concerning in Native American populations, among whom 38·1 % of adults aged 18 years and over are classified as obese^(2)^, and the consumption of SSB is up to 34 % more likely compared with US non-Hispanic whites^(3)^. Nation- and statewide intervention efforts to decrease the intake of energetic beverages do not always consider the unique cultural and lifestyle factors of Native American populations, such as distinctive core cultural values, heritage and history^(4)^. Therefore, culturally appropriate interventions are needed to support healthy food environments and behaviours that reduce energetic beverage intake.

SSB include soft drinks (soda, pop), fruit drinks, energy drinks and sweetened flavoured waters. Research has shown that SSB can increase glycaemic load and lead to insulin resistance, β-cell dysfunction and inflammation and risk factors not only for obesity but also for metabolic syndrome and type 2 diabetes^(5,6)^. Additionally, SSB are thought to increase risk for overweight and obesity due to incomplete compensation for total energy at subsequent meals throughout the day^(5,7,8)^.

In Native American populations, SSB have been identified as a leading source of energy for young adult women of the Hualapai tribe^(9)^ and adults of the Havasupai tribe^(10)^. The Navajo Health and Nutrition Survey identified soft drinks as contributing 7 % of total energy intake for adult respondents^(11)^, and high rates of SSB consumption and lower water consumption have also been reported in Alaska Native adults and children living in rural Alaska^(3)^, where Alaska Native adults are more than three times as likely as non-Hispanic whites to consume three or more SSB per day ^(12)^. Canadian First Nations and Inuit populations also demonstrate high intake of SSB^(13)^ as well as a negative association between SSB consumption, BMI and impaired fasting glucose^(14)^.

Other energetically dense beverages, such as whole milk, may also contribute to overweight and obesity in Native American populations. Whole milk specifically has been identified as one of the most frequently consumed beverages in several Native American populations^(15)^, indicating its substantial overall contribution to energetic intake. Low-fat alternatives, including 2 % and skim milk, have been associated with decreased risk of metabolic syndrome^(16)^, type 2 diabetes^(17)^ and atherogenic lipid profiles^(18)^.

Interventions focused on improving overall dietary intake^(19–26)^ have taken place in Native American and First Nations communities, but success has varied. With the exception of Healthy Foods North, a multi-level, multi-component (MLMC) intervention in First Nations communities^(23)^, existing interventions have not consistently targeted beverage intake, nor have they addressed factors specific to Indigenous populations or been developed in partnership with Native American communities to include culturally appropriate messages and validated data collection methods and tools. Additionally, these interventions operated at selected levels within communities and did not utilise a comprehensive MLMC approach to influence the food environment at multiple levels, allowing for maximal intervention exposure and reinforcement of the behaviour(s) being promoted. An MLMC approach may be especially impactful in rural Native American communities, where food stores and worksites are few^(27)^, and there is high potential for interventions to saturate the local environment.

Finally, some interventions have focused on school children^(28)^, yet there is little evidence from programmes utilising a comprehensive intervention targeting both adults and schoolchildren, with children acting as change agents. Outcomes may differ substantially between child and adult participants; while a school setting may provide the most efficient means to prevent or reduce risk behaviours due to the potential to reach large segments of the population^(29,30)^, schoolchildren are still limited in their ability to purchase and consume healthy foods and beverages due to their reliance on adult caregivers. Adults, on the other hand, exercise greater purchasing and decision-making power, yet may be harder to reach throughout various community levels (i.e., may only go food shopping once a week or may be employed outside of the community). The health promotion efforts at schools could have a significant impact on household food choices and potential for the adoption of healthy behaviours in adults in the future, yet this has not been fully explored in the literature.

Lack of conclusively successful interventions in adult Native Americans and the scarcity of MLMC programmes in the literature demonstrate the need for additional research in this population. The Obesity Prevention and Evaluation of InterVention Effectiveness in NaTive North Americans (OPREVENT) intervention was developed to address this gap. The theory-based, culturally adapted programme sought to address dietary intake using a MLMC design targeting multiple levels within the environment that have shown to have potential for success in the literature, including food stores, worksites, schools and media^(27)^. While the objective of the overall intervention was to improve nutritional intake, the food purchasing environment and physical activity (PA), the objective of the current analysis was to evaluate the impact of OPREVENT on beverage intake in Native American adults. It was hypothesised that respondents in Intervention communities would decrease the consumption of discouraged beverages from pre-intervention to post-intervention as compared with respondents in the Comparison communities.

## Methods

The development and design of the OPREVENT programme have been described in detail elsewhere^(31)^. The following provides a brief overview.

### Setting

To be eligible for OPREVENT, tribal communities were required to have an on-reservation population of >500, at least one on-reservation school, at least one on-reservation food store and at least one worksite with at least five tribal member employees. These populations and venue criteria increased the likelihood of pre-existing resources, ensured that there were tribally operated venues in which to implement the intervention and guaranteed a degree of programme sustainability beyond the involvement of the Baltimore, Maryland, USA-based research team. Eight tribal communities were selected (reduced to five before the formative phase due to budgetary constraints). Three were in the Southwestern United States, and two were in the upper Midwestern United States. Community characteristics have been reported elsewhere^(31)^.

### Intervention design

The OPREVENT intervention was rooted in social cognitive theory as well as the social ecological model^(31)^. Together, both theories emphasise the individual’s ability to influence the environment to support the desired behaviour change and the importance of working within multiple ecological levels to build capacity for collective action^(31)^. These principles helped in identifying collaborative partnerships within the communities as well as shaping OPREVENT content and messaging.

The design of OPREVENT used a community-driven approach and was informed by an extensive formative phase, consisting of participant observation, focus groups, in-depth interviews and community workshops within four of the five participating communities (one of the five participating communities chose not to participate in the formative phase). Detailed methodology and qualitative findings are described elsewhere^(32–34)^. Specific to the current analysis, community member and stakeholder participants in the formative phase identified ‘problem foods, beverages and behaviors,’ or foods, beverages and behaviours that they believed were problems within their communities and the main contributors to poor health among community members. Participants also identified settings within their communities where they believed intervention efforts would be most impactful; for example, worksites where many community members were employed. This provided the basis for the development of the intervention. The result was a community-centred MLMC obesity intervention programme with food store, worksite, school and media components that discouraged these problem foods, beverages and behaviours by emphasising traditional foods and PA and utilised culturally informed and appropriate materials and evaluation methods. It was implemented in Intervention communities by local, Native American interventionists in five phases over 14 months beginning in the summer of 2012, with each phase focused on specific target foods and beverages, PA and/or associated food-related behaviours such as cooking or meal planning.

**Fig. 1:**
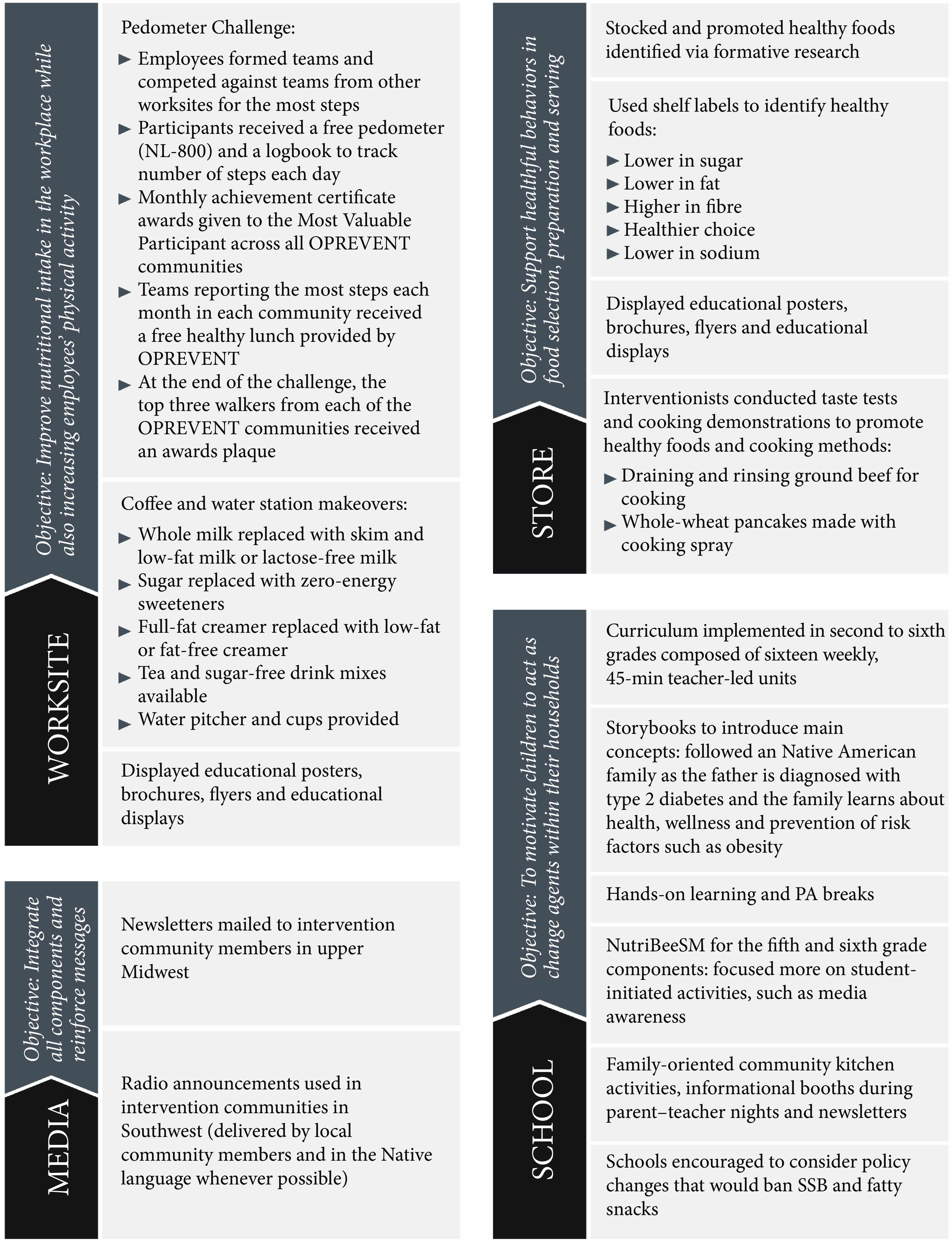
Obesity Prevention and Evaluation of InterVention Effectiveness in NaTive North Americans (OPREVENT) intervention components. PA, physical activity

Intervention components are described in detail in Fig. 1. The OPREVENT intervention sought to improve dietary intake and PA via education, promotional activities and partnerships with community institutions to support a healthier food environment while also providing opportunities for training, local capacity building and employment to help ensure sustainability. Acceptable and affordable healthier alternatives to the problem foods and beverages identified in the formative phase were promoted throughout the intervention, while the problem foods and beverages were discouraged. Emphasis was placed on working with participating stores to stock an array of sugar-free, low-sugar or low-energy beverage items including water, zero-energy flavoured water, low-fat milk and milk substitutes and non-sugar drink mixes. Although no price intervention was implemented due to managerial constraints placed on store owners, store owners were encouraged to improve visibility of healthier beverage options (i.e., by placing healthier beverages at eye level or moving to point of purchase). Dietary messages specific to beverages included reducing the intake of energetically dense beverages by replacing them with sugar-free alternatives and drinking more water. Interventionists conducted interactive taste tests utilising promoted beverages at participating food stores and assisted shoppers in finding the items on the shelves. Participating worksites and schools were encouraged to adopt policies that prohibited the sale of SSB on site – for example, in vending machines – and encouraged employees and students to choose healthier alternatives. Coffee and water station makeovers were implemented at worksites, replacing unhealthy items such as sugar with healthier alternatives identified in the formative phase such as zero-energy sweeteners. A comprehensive school curriculum was implemented in grades 2–6 and included in-class activities as well as take-home assignments to be completed as a family, such as creating a grocery list of healthy beverages together. All materials and messages were developed in partnership with community members and stakeholders, with artwork done by local artists, to ensure cultural acceptance.

### Research design

To evaluate OPREVENT, a year-long community-randomised controlled trial was conducted in the five participating Native American communities. The five communities were randomised to Intervention (*n* 3) or Comparison (*n* 2). One upper Midwestern community and two Southwestern communities were randomised to Intervention, and one supper Midwestern community and one Southwestern community were randomised to Comparison.

Intervention communities received the OPREVENT programme for 14 months beginning in the summer of 2012; Comparison communities also received the programme after completion of the study and post-intervention data collection. Food stores, worksites and schools were recruited from each community and received intervention materials and support from the Johns Hopkins Bloomberg School of Public Health study team in return for volunteering to participate.

Households in each community were randomly selected from tribal lists. Within each household, one adult between the ages of 18 and 75 years old who had been living there for at least 30 d was randomly selected. Other inclusion criteria included English-speaking, self-identified community member and the primary food shopper and/or preparer for the household. Exclusion criteria included currently pregnant or nursing women. If the adult was eligible but declined to participate, enrollment continued with the next household on the list. The aim was to enrol eighty-five adults pre-intervention from each community, for a total *n* 424. This resulted in *α* = 0·05 and power = 80 % to detect a decrease of 1338·88 kJ in total energy intake.

Data collection was conducted in both Intervention and Comparison communities at baseline (spring 2011–2012) and post-intervention (fall 2013/winter 2014). Comparison communities received delayed intervention at the conclusion of the trial.

### Data collection instruments and outcomes

Data collection was done pre-intervention and post-intervention. Interviews were done by trained local Native American data collectors and took approximately 90–120 min to complete. Respondents received $40 gift cards to Wal-Mart after each interview session.

**Fig. 2:**
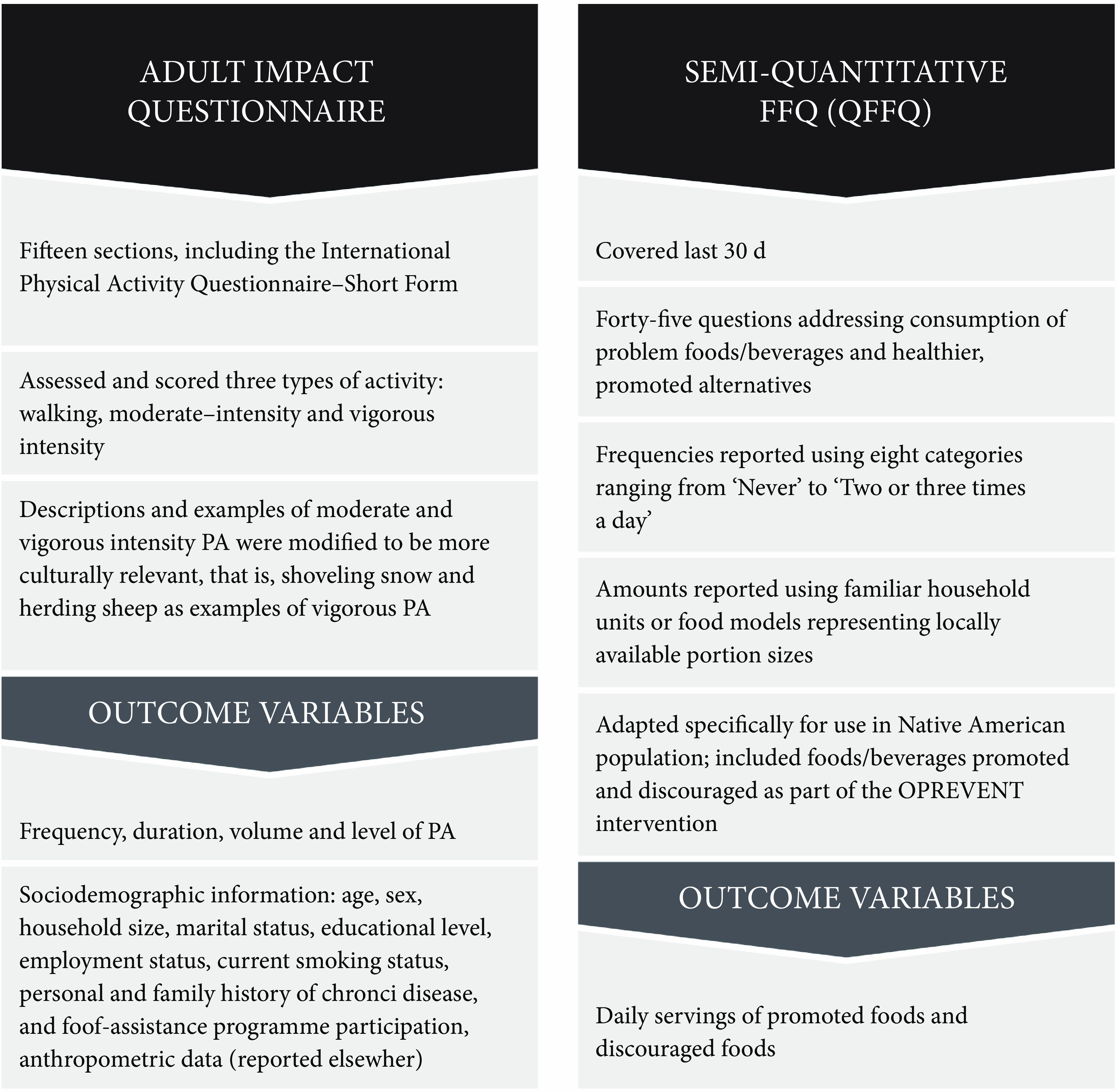
Obesity Prevention and Evaluation of InterVention Effectiveness in NaTive North Americans (OPREVENT) data collection instruments. PA, physical activity

Data collection instruments are described in Fig. 2. Instruments were administered via interview and included the adult impact questionnaire and a brief semi-quantitative FFQ which provide the basis for the current analysis. The adult impact questionnaire was adapted from formative research findings and also previous work conducted in Native American and First Nations settings^(19,35–37)^. Data from the adult impact questionnaire reported in the current analysis include demographic information and anthropometric measurements. The FFQ was adapted from the previous work in Native American communities^(38,39)^. Only beverage-specific questions were used for the current analysis.

The FFQ was administered at both pre-intervention and post-intervention to all respondents in both Intervention and Comparison communities. Respondents were asked the following: ‘How often during the last 30 days did you USUALLY eat the following foods and how much do you USUALLY eat at one time?’ Choices for the frequency of consumption ranged from ‘never’ to ‘2 or more times per day’, and choices for portion size were based on commonly consumed portion sizes within the communities, as identified via formative research. The nine beverage-specific items included (1) milk (whole) (including Lactaid® whole), (2) milk (2 %) (including Lactaid® 2 %), (3) milk (1 %) or skim (including Lactaid® 1 % or skim), (4) any regular soda, including energy drinks, Coke®, Mountain Dew®, Pepsi®, Monster®, Red Bull®, (5) 100 % juice (apple, orange, grape, Juicy Juice®), (6) sugary beverages such as sweetened iced tea, lemonade, fruit punch, Gatorade®, fruit cocktail, cranberry juice, Sunny D® or other sugary drinks that are not 100 % juices or soda, (7) sugar-free drinks (including diet soda, Crystal Light®, unsweetened or diet tea), (8) water (including bottled or tap water) and (9) alcohol (like beer, wine, liquor). Standard serving sizes were 20 oz for items 4 and 7 and 8 oz for all other items. Example beverages listed for each item were chosen by community members during the formative phase as beverages that were consumed frequently and would be recognised by respondents. Items 1, 4 and 6 were discouraged, while items 2, 3, 5, 7 and 8 were promoted as healthier alternatives. Item 9, alcohol, was not specifically addressed in intervention messaging due to the sensitive nature of this topic and the fact that alcohol education programmes were already in place within participating communities. However, due to community identification as a problem beverage during the formative phase and tribal requests for data related to consumption, it was still included in the current analysis. Coffee was not included as a separate item or as a specific example as it was not identified as a problem beverage with sufficient frequency during the formative phase. Flavoured milks, while higher in sugar than unflavoured varieties, were not identified as a commonly consumed problem beverage during the formative phase and were classified on the FFQ by fat content.

### Data management

All data were entered into Microsoft Access databases and Microsoft Excel spreadsheets (Microsoft Corporation) and then exported to Stata software, version 12.1 (StataCorp) for analysis. To prepare the FFQ data, reported portion sizes and 30-d frequencies were used to calculate total servings of each beverage item. Daily servings were calculated by dividing the 30-d total by 30 and were analysed as the primary outcome for the current analysis.

### Analysis

Descriptive analyses comparing pre-intervention characteristics of the Intervention and Comparison communities were conducted. Student’s *t* tests were used for normally distributed continuous variables, non-parametric Wilcoxon–Mann–Whitney tests were used for non-normal continuous variables and *χ* ^2^ tests were used for categorical and dichotomous variables. These tests were also used to compare respondents who were lost to follow-up to those remaining in the study at the end of the intervention.

Intervention impacts were assessed using the difference-in-differences (DiD) method^(40)^. Regression analyses were performed on each variable of interest with time, intervention assignment and the interaction term time × intervention as covariates, with clustering at the community level to account for potentially decreased between-person variation among individuals living in the same communities.

The primary outcome for the analysis was daily servings, as this was felt to be a more meaningful and actionable measure for community members and to align with messaging from both the OPREVENT intervention and national health initiatives, such as MyPlate and the Dietary Guidelines for Americans, that also make recommendations based on daily servings. Daily servings of promoted beverages and discouraged beverages were analysed using the DiD method to determine if there were greater positive changes in respondents living in Intervention communities compared with those from Comparison communities. Sub-analyses were then performed to determine whether the changes in dietary outcomes differed by gender, since it was anticipated that females would be more exposed to the intervention due to their traditional role as primary food shoppers and preparers in this population. *α* values were set to 0·05.

## Results

Data were collected on 422 respondents pre-intervention and 299 respondents post-intervention, for a 71 % retention in the Intervention communities and 69 % retention in the Comparison communities. Respondents lost to follow-up due to incomplete post-intervention data (*n* 123) were significantly younger (41·5 *v*. 44·5 years; *P* < 0·05), less likely to receive benefits from women, infants and children (17·6 *v*. 29·2 %; *P* < 0·01) and less likely to receive commodity foods (4·9 *v*. 13·8 %; *P* < 0·01) than those who completed the study.

The comparisons of pre-intervention characteristics are shown in Table 1. The evaluation sample was predominantly female (70·8 %). The prevalence of obesity, as indicated by BMI ≥ 30 kg/m^2^, was high (55·2 %). There were significant differences between the Intervention and Comparison communities. Respondents in the Intervention communities were more likely to be the primary food preparer within their household (95·0 *v*. 88·0 %; *P* < 0·05), have a tech school degree (8·9 *v*. 2·6 %; *P* < 0·05) or some college education (33·0 *v*. 16·2 %; *P* < 0·05), have a smaller household size (3·5 ± 2·0 *v*. 4·3 ± 2·4; *P* < 0·05) and participate in commodity food programmes and senior centre food programmes (18·2 *v*. 6·9 % and 15·5 *v*. 3·5 %, respectively; *P* < 0·05). Intervention community respondents were less likely to participate in the Supplemental Nutrition Assistance Program (43·1 *v*. 61·2 %; *P* < 0·05). We observed similar lost to follow-up for Intervention and Comparison communities. No adverse events were reported for respondents from either group.

**Table 1:** Sociodemographic characteristics of the Obesity Prevention and Evaluation of InterVention Effectiveness in NaTive North Americans evaluation sample (*n* 299), pre-intervention

Characteristics	Full sample (*n* 422)		Intervention (*n* 182)		Comparison (*n* 117)	
Female (%)	70·8		69·6		72·7	
Age (years)						
Mean		44·5		45·2		43·3
sd		13·5		14·0		12·6
Primary food shopper and/or preparer in household	92·3		95·0		88·0	
Highest completed education (%)						
High school	25·3		22·9		29·1	
Tech school	6·4		8·9		2·6*	
Some college	26·4		33·0		16·2*	
College	2·4		2·2		2·6	
Married (%)	32·7		31·3		34·8	
Household size (*n*)						
Mean		3·8		3·5		4·3*
sd		2·2		2·0		2·4
Current smoker (%)	28·0		24·3		34·0	
History of disease (%)						
Obesity	44·8		47·2		41·1	
Heart disease	8·6		8·5		8·8	
High blood pressure	31·7		32·4		30·7	
Type 2 diabetes	22·4		20·5		25·4	
Not employed (%)	42·5		41·2		44·4	
Participation in food assistance programme (%)						
WIC	17·6		14·4		22·4	
SNAP	50·2		43·1		61·2*	
Commodity	13·8		18·2		6·9*	
Senior centre	10·8		15·5		3·5*	
Food bank	7·7		5·5		11·2	
BMI (kg/m^2^)						
Mean		32·2		32·3		32·0
sd		7·9		7·7		8·1
Overweight† (%)	29·0		28·9		29·1	
Obese‡ (%)	55·2		57·2		52·1	

WIC, women, infants and children; SNAP, Supplemental Nutrition Assistance Program.

* Significantly different between intervention and comparison communities at the *P* < 0·05 significance level.

† BMI ≥ 25 and ≤29·9 kg/m^2^.

‡ BMI ≥ 30 kg/m^2^.

### Discouraged beverages

Table 2 summarises the DiD for discouraged beverages. Regular soda was the most frequently consumed discouraged beverage in both the Intervention and Comparison communities pre-intervention and post-intervention. Daily servings of whole milk, sugary drinks and alcohol remained approximately the same from pre-intervention to post-intervention in both the Intervention and Comparison communities; no significant DiD was observed for any of these three discouraged beverages. Daily servings of regular soda decreased in both Intervention and Comparison communities, with a greater decrease in Intervention (6 *v*. 2 oz). The DiD for regular soda was significant (−0·3; *P* < 0·05). Female respondents had significantly greater DiD for daily servings of regular soda compared with male (−0·4 *v*. −0·1, respectively; *P* < 0·05) (data not shown).

**Table 2:** Daily servings with se of discouraged beverages: baseline, post-intervention and difference-in-differences (DiD) in Intervention (I) and Comparison (C) Obesity Prevention and Evaluation of InterVention Effectiveness in NaTive North Americans communities

	Pre-intervention						Post-intervention						DiD	se_DiD_	*P*
	C	se_C_	I	se_I_	Difference	se_Diff_	C	se_C_	I	se_I_	Difference	SE_Diff_			
Whole milk*	0·2	0·5	0·1	0·2	−0·1	0·1	0·1	0·3	0·1	0·3	0·0	0·1	0·1	0·0	0·076
Regular soda†	0·7	0·9	0·7	1·3	0·1	0·1	0·6	1·1	0·4	0·7	−0·2	0·1	−0·3	0·1	0·038
Sugary drinks‡	0·3	0·5	0·2	0·5	0·0	0·0	0·2	0·5	0·3	0·5	0·0	0·1	0·0	0·1	0·981
Alcohol§	0·1	0·4	0·2	1·0	0·2	0·2	0·1	0·3	0·2	0·8	0·2	0·2	−0·0	0·0	0·495

Diff, difference.

* 8 oz milk (whole) (including Lactaid® whole).

† 20 oz regular soda, energy drinks, Coke®, Mountain Dew®, Pepsi®, Monster®, Red Bull®.

‡ 8 oz sweetened iced tea, lemonade, fruit punch, Gatorade®, fruit cocktail, cranberry juice, Sunny D® or other sugary beverages that are not 100 % juices or soda.

§ 8 oz beer, wine, liquor.

### Promoted beverages

The DiD for promoted beverages is shown in Table 3. Water was the most frequently consumed promoted beverage in both Intervention and Comparison communities pre-intervention and post-intervention. Daily servings of 2 % milk, 1 % or skim milk, 100 % juice, sugar-free drinks and water remained the same from pre-intervention to post-intervention in both Intervention and Comparison communities. There were no significant DiD estimates observed in the evaluation sample.

**Table 3:** Daily servings with se of Promoted beverages: pre-intervention, post-intervention, and difference-in-differences (DiD) in Intervention (I) and Comparison (C) Obesity Prevention and Evaluation of InterVention Effectiveness in NaTive North Americans communities

	Pre-intervention						Post-intervention						DiD	se_DiD_	*P*
	C	se_C_	I	se_I_	Difference	se_Diff_	C	se_C_	I	se_I_	Difference	se_Diff_			
2 % milk*	0·3	0·6	0·2	0·4	−0·1	0·1	0·3	0·5	0·2	0·4	−0·1	0·1	0·0	0·0	0·893
1 % or skim milk†	0·1	0·3	0·1	0·3	0·0	0·1	0·1	0·3	0·1	0·3	0·0	0·0	0·02	0·0	0·460
100 % juice‡	0·3	0·4	0·2	0·4	−0·0	0·1	0·3	0·4	0·2	0·3	−0·1	0·1	0·04	0·0	0·451
Sugar-free drinks§	0·6	1·0	0·6	0·9	0·1	0·2	0·5	0·9	0·7	0·8	0·3	0·2	0·2	0·1	0·112
Water‖	1·6	1·6	2·0	2·4	0·4	0·2	1·7	1·4	1·8	1·6	0·1	0·2	−0·3	0·3	0·331

Diff, difference.

* 8 oz milk (2 %) (including Lactaid® 2 %).

† 8 oz milk (1 %) or skim (including Lactaid® 1 % or skim).

‡ 8 oz 100 % juice (apple, orange, grape, Juicy Juice®).

§ 20 oz diet soda, Crystal Light®, unsweetened or diet tea.

‖ 8 oz bottled or tap.

## Discussion

This is the first study to examine changes in beverage intake resulting from an MLMC obesity intervention in Native American adults. We found that Native American adults living in Intervention communities significantly decreased daily consumption of regular soda in comparison with Native American adults living in Comparison communities but did not increase the intake of healthier beverages promoted by the intervention.

In Intervention communities, daily servings of regular soda decreased by approximately 0·3 servings, or 6 oz. An average 20 oz serving of regular soda, the standard size reported by our evaluation sample, contains 1046 kJ all from added sugars. A decrease of 6 oz represents a decrease of approximately 313·8 kJ /d. All other variables held constant, a daily decrease of 313·8 kJ could result in a weight loss of just under 10 pounds/year. For the OPREVENT evaluation sample, with an average weight of 190 pounds, this reduction in soda consumption alone could represent a 5 % weight loss, which is considered clinically important. Preliminary analysis of changes to the food components of dietary intake assessed by the FFQ did not show any significant changes in the intake of other sugary items, such as sweets and candies (data not shown), indicating that OPREVENT respondents did not compensate for total energy intake. Therefore, it is reasonable to believe that if respondents were to engage in other healthy behaviours, including those encouraged by OPREVENT, a clinically significant weight loss of 10 % could be possible.

Because of the well-established positive association between added sugar intake and obesity and other diet-related chronic diseases^(5,6)^, the American Heart Association suggests limiting added sugars to no more than half of the daily discretionary energy allowance (approximately 418·4 kJ/d for women and 627·6 kJ/d for men)^(41)^, and the USDA’s 2015 Dietary Guidelines recommend that added sugars not exceed 10 % of total energy intake each day^(42)^. Post-intervention, the Intervention respondents in the OPREVENT evaluation sample were consuming an average of 376·56 kJ/d from regular soda, which is within range of the American Heart Association’s guideline for both men and women.

There was a small, insignificant decrease in daily servings of regular soda in the Comparison communities. Contamination between Intervention and Comparison community respondents was possible, as Comparison community members may have had friends and family in Intervention communities with whom they visited and therefore may have been exposed to intervention content. Additionally, the Healthy Diné Nation Act was implemented within the same year as OPREVENT throughout the Navajo Nation and would have included both Intervention and Comparison communities. This Act imposed a 2 % tax on all SSB (as well as foods high in salt, fat and/or sugar). Like OPREVENT, the focus was on all SSB, but the media tended to refer to the tax as the ‘junk food and soda tax’, placing the emphasis on soda alone. This may have caused respondents in Comparison communities to reduce soda intake and further reinforced the decreased soda messaging in Intervention communities.

The change in consumption of other SSB was not significantly different between Intervention and Comparison communities. Although OPREVENT’s messages did include decreasing all SSB, such as sweet tea, energy drinks and high-sugar powdered drink mixes, the focus was on regular soda. Community members emphasised regular soda as an unhealthy ‘problem food’ during formative work. As such, discouragement of regular sodas may have overshadowed that of other SSB.

The change in consumption of healthy beverage alternatives was also not significantly different between Intervention and Comparison communities. Despite OPREVENT’s messages encouraging the consumption of healthier alternatives, this was not reflected in the data.

Females had significantly greater change in daily servings of regular soda compared with males. Inclusion criteria for the OPREVENT evaluation sample required that respondents to be the primary food shopper or preparer in their household, a role traditionally fulfilled by females in the OPREVENT communities. If females were primarily responsible for food shopping, it is likely that they were more exposed to the intervention materials in the food stores, leading to greater changes.

Other adult obesity interventions promoting healthy dietary intake in Native American adults have shown similar results in decreased consumption of soda and other SSB. The Zuni Diabetes Prevention Program was a 6-year intervention that took place on the Zuni Indian reservation in Western New Mexico and targeted multiple risk factors for diabetes and obesity, including low PA and high consumption of SSB^(27)^. A midpoint evaluation of the programme revealed a significant decrease in the consumption of SSB, and while the target population was adolescents rather than adults, the findings from OPREVENT confirm these trends previously reported in the literature from the Zuni Diabetes Prevention Program. Healthy Foods North was a MLMC obesity intervention in a remote First Nations community in Canada that also discouraged certain unhealthy foods and beverages, similar to those discouraged in OPREVENT^(35)^. Researchers observed a significant decrease in the consumption of unhealthy drinks (including regular pop, sweetened juice and sweetened drinks such as Tang^®^, fruit punch and Kool-Aid^®^) in the intervention group, going from an average intake of 754 to 587 g/d^(35)^. This is equivalent to a decrease from 26·6 to 20·7 oz/d, or 5·9 oz, which is similar to the results obtained in OPREVENT for regular soda alone. A similar study also conducted within an FN reserve reported decreased intake of unhealthy drinks as well^(43)^. Finally, the Shape Up Somerville study found that children exposed to the intervention decreased the consumption of SSB by more than 12 oz/week but did not observe any change in fruit and vegetable consumption^(44)^. The intervention was a 2-year community-based trial within multiple components, much like OPREVENT, and although the target population was children and not Native American adults, the results corroborate our findings and show the potential of MLMC interventions to influence the consumption of a dietary component associated with obesity.

### Limitations

The brief FFQ was not meant to capture the majority of foods and beverages consumed by community, but rather only the promoted and discouraged foods and beverages. As such, we were unable to assess the contribution of the promoted and discouraged beverages to total dietary intake. This may have yielded a more complex analysis and a richer understanding of the dietary changes that may have taken place. Also because of this, we were unable to accurately estimate energy and nutrient intakes, which may have revealed other changes. Additionally, FFQ may be prone to other limitations such as response and recall bias, systematic error and random error in measurement. Participants must accurately recall portions and frequency of consumption of many food and beverage items, relying on cognitively complex memory and averaging tasks. They may also report what they believe to be a socially desirable response, or report their intake differently at two different times, all of which may contribute to imperfect estimations.

Overall generalisability is limited. The inclusion criteria required that respondents be either the primary food shopper or food preparer within their household, a role typically filled by women in the OPREVENT communities. Therefore, the evaluation sample was not representative of all individuals in the five communities, nor of the general adult Native American population. We also experienced a substantial loss to follow-up. Additionally, although choosing to report daily servings as the primary outcome of interest was important for our tribal partners, it does limit the ability to compare with existing literature.

Finally, an inherent limitation of MLMC interventions is the difficulty in assessing which components contribute to intervention outcomes and which ones do not. We were unable to sufficiently assess this in the present intervention and therefore are limited in ability to draw conclusions regarding the effectiveness of each component.

### Implications for research and practice

In summary, we demonstrated that a MLMC obesity intervention programme in Native American communities significantly decreased the consumption of regular soda as compared with those living in Control communities. The implications of these findings are far reaching. SSB contribute significantly to energy intake in Indigenous populations worldwide^(9–14,45–47)^. As with Native American adults, these populations are disproportionately affected by obesity and type 2 diabetes^(48–53)^. Decreasing consumption of SSB via MLMC interventions may positively impact risk for these conditions. These findings add to the growing literature of intervention trials seeking to improve dietary intake as a means of addressing obesity in these populations. Future research efforts should utilise MLMC design that includes efforts to increase overall retention and exposure, ensuring that intervention messages clearly identify foods, beverages and behaviours to be both promoted and discouraged and consider designs which permit testing of different components alone or in combination.
